# Raman Stable Isotope Probing of Bacteria in Visible and Deep UV-Ranges

**DOI:** 10.3390/life11101003

**Published:** 2021-09-24

**Authors:** Georgette Azemtsop Matanfack, Aikaterini Pistiki, Petra Rösch, Jürgen Popp

**Affiliations:** 1 Institute of Physical Chemistry and Abbe Center of Photonics (IPC), Friedrich-Schiller-University Jena, Helmholtzweg 4, 07743 Jena, Germany; georgette.azemtsop@uni-jena.de (G.A.M.); aikaterini.pistiki@uni-jena.de (A.P.); juergen.popp@uni-jena.de (J.P.); 2 Leibniz Institute of Photonic Technology (Leibniz-IPHT), Member of Leibniz Research Alliance “Health Technologies”, Albert-Einstein-Straße 9, 07745 Jena, Germany; 3 Research Campus Infectognostics e.v., 07743 Jena, Germany

**Keywords:** Raman microspectroscopy, Raman UV-resonance, bacterial cells, DNA labeling, metabolic activity

## Abstract

Raman stable isotope probing (Raman-SIP) is an excellent technique that can be used to access the overall metabolism of microorganisms. Recent studies have mainly used an excitation wavelength in the visible range to characterize isotopically labeled bacteria. In this work, we used UV resonance Raman spectroscopy (UVRR) to evaluate the spectral red-shifts caused by the uptake of isotopes (^13^C, ^15^N, ^2^H(D) and ^18^O) in *E. coli* cells. Moreover, we present a new approach based on the extraction of labeled DNA in combination with UVRR to identify metabolically active cells. The proof-of-principle study on *E. coli* revealed heterogeneities in the Raman features of both the bacterial cells and the extracted DNA after labeling with ^13^C, ^15^N, and D. The wavelength of choice for studying ^18^O- and deuterium-labeled cells is 532 nm is, while ^13^C-labeled cells can be investigated with visible and deep UV wavelengths. However, ^15^N-labeled cells are best studied at the excitation wavelength of 244 nm since nucleic acids are in resonance at this wavelength. These results highlight the potential of the presented approach to identify active bacterial cells. This work can serve as a basis for the development of new techniques for the rapid and efficient detection of active bacteria cells without the need for a cultivation step.

## 1. Introduction

The metabolic profile of bacteria changes due to different environmental conditions [1,2]. Understanding the dynamics of bacterial metabolism is the key to elucidating biological processes [1,2,3,4]. The identification of metabolically active bacterial cells is of high relevance in clinical diagnosis [5,6,7,8], industrial biotechnology, and microbial ecology [1,9,10,11,12]. The standard method for identifying metabolically active cells in biomedical samples involves the use of a gas sensor to monitor the increase in CO_2_ concentration in bacterial cultures [13,14]. However, this conventional method is culture-dependent and requires a long operating time, making it non-ideal for online analysis [14]. Therefore, continuous efforts are being made to develop culture-independent techniques for the rapid and reliable identification and characterization of metabolically active cells [1,2,5,6,7,8,9,10,11,12,15,16,17,18,19,20,21,22,23,24].

Raman microspectroscopy is a valuable tool that can be used to analyze the molecular composition of bacterial cells [25]. Since it is a phenotypic technique, changes in the molecular structure of bacteria are reflected in the Raman spectra [1,26]. Raman microspectroscopy combined with stable isotope probing (Raman stable isotope probing) allows the analysis of the metabolism of bacterial cells. The incorporation of stable isotopes such as ^18^O, ^13^C, ^15^N, and ^2^H (D) into the cellular biomass leads to a red-shift in the Raman spectra of bacteria [4,11,16,18,27,28]. Hence, stable isotopes can be used as tracers to visualize the dynamics of bacterial metabolism. Recently, Raman-SIP has been intensively applied in order to investigate the metabolic activity of bacteria in their natural habitats such as soil systems [11,12,21,29], water [9,17], and colon [6,24]. The Raman-SIP of bacteria has been previously shown using laser excitation in the visible range [7,10,16,27]. Recently, the characterization of ^18^O-labeled bacteria in the deep-UV range has been explored. However, the detection of ^13^C, ^15^N, and D in the deep-UV range has never been reported. In UV resonance Raman spectroscopy (UVRR), the excitation wavelength matches the electronic absorption of molecules containing an aromatic ring, such as aromatic amino acids, purine, and pyrimidine bases of nucleic acids. Therefore, the Raman signals of these biomolecules are selectively enhanced compared to all of the other cellular components. Due to this benefit, Raman-spectroscopic investigation of bacteria in the UV excitation range has been shown extensively in the past [25,30,31,32]. 

Herein, we studied the assimilation of stable isotopes (^18^O, ^13^C, ^15^N, and D) into bacterial biomass using UVRR. The spectral variations caused by the isotopes were examined using the characteristic red-shifts of Raman bands. In addition, we evaluated the sensitivity of detecting metabolically active cells by tracing isotope incorporation into the DNA material extracted from isotopically labeled bacteria. We demonstrated the applicability of this approach using an excitation wavelength in the deep-UV range compared to a visible excitation wavelength. The present study highlights the potential of UVRR and provides the basis for a DNA-based method for the automatic identification of metabolically active bacteria.

## 2. Materials and Methods

### 2.1. Bacterial Culture and Sample Preparation

The chemicals used for this study were purchased from Sigma Aldrich (Munich, Germany) unless otherwise specified. The investigation was performed on the model system *E. coli* DSM 501 pre-grown on an M9 minimal medium agar plate for 24 h at 37 °C. A few colonies were inoculated into 15 mL of M9 minimal medium (1 g/L ammonium chloride, 6 g/L disodium hydrogen phosphate, 3 g/L potassium dihydrogen phosphate, 0.5 g/L sodium chloride, 2 mL/L 1 M magnesium sulfate solution, and 20 mL/L 20% glucose solution, pH 7.4) and were incubated for 24 h at 37 °C with 120 rpm shaking. The medium composition varied with the addition of each isotope. The corresponding substrates were replaced by the heavier substrates. For the addition of ^13^C, ^15^N, ^2^H (D), and ^18^O, the components ^13^C_6_-glucose (99 atom %), ^15^NH_4_Cl (98 atom %), D_12_-glucose (99 atom %), and H_2_^18^O (97 atom %) were used, respectively. Three biological replicates were prepared for each sample on different days. Following incubation, 1 mL of each bacterial culture was centrifuged at 5000× *g* for 5 min, washed three times with sterile deionized water to remove the medium, and resuspended in 1 mL of deionized water. Subsequently, 10 µL of the final suspension was allowed to air dry on a nickel foil and was used for single-cell Raman measurements in the visible range (532 nm).

For bulk Raman measurements in the deep-UV range (244 nm), 5 mL of the bacterial culture were washed analogous to the samples for the measurements with 532 nm excitation. The resulting pellet was resuspended in 30 µL distilled water and was then placed onto fused silica slides and left to air-dry prior to measurement. 

### 2.2. DNA Extraction

Nucleic acids were extracted from 15 mL of each of *E. coli* culture using the QIAamp DNA mini kit (Qiagen GmbH, Germany, cat: 51304), following the manufacturer’s instructions. Two biological replicates were prepared on different days. DNA elution was conducted in nuclease-free, molecular grade water (Roth, Karlsruhe, Germany Cat: T143). The DNA yield was quantified using the Quibit 1XdsDNA HS Assay kit (Invitrogen, Waltham, MA, USA cat: Q33230) and is summarized in Table 1 for each measured biological replicate.

For the 532 nm excitation, a drop of the isolated DNA was placed on Ni-foil and was left to air-dry under the bench. For the UVRR, 30 μL of solution was placed onto fused silica slides and was measured directly. Single-strand 10mer oligonucleotides were purchased from Sigma-Aldrich with the following sequences: 5′-AAAAAAAAAA-3′, 5′-CCCCCCCCCC-3′, 5′-GGGGGGGGGG-3′, and 5′-TTTTTTTTTT-3′, similar to a previous work [33]. Oligonucleotides were received in a lyophilized form and were reconstituted in nuclease-free, molecular grade water (Roth) to a concentration of 100 mM. One biological replicate was measured.

### 2.3. Raman Measurements

A Raman microscope (Bio Particle Explorer; rap.ID Particle Systems GmbH, Berlin, Germany) equipped with a 532 nm solid-state frequency-doubled Nd:YAG laser (LCM-S-111-NNP25; Laser-export Co. Ltd., Moscow, Russia) was used. The Raman spectra were collected using a 100× air objective (MPLFLN-BD, NA = 0.90, Olympus Corporation, Tokyo, Japan) with a spot size of below 1 µm. The exposure time per single Raman spectrum was 15 s, and the laser power was 9 mW on the sample. The backscattered Raman light was dispersed by a single-stage monochromator (HE 532, Horiba Jobin Yvon, Munich, Germany) equipped with a 920 lines/mm grating and was finally detected by a thermoelectrically cooled charge-coupled device (CCD) camera (DV401-BV; Andor Technology, Belfast, Northern Ireland) with a spectral resolution of about 8 cm^−1^. 

For the bulk biomass measurements in the deep UV region, a Raman microscope (HR800, Horiba/Jobin-Yvon, Bensheim, Germany) was used. The system was equipped with a 244 nm frequency-doubled Argon-ion laser (Innova 300, FReD, Coherent, Dieburg, Germany) and a focal length of 800 mm. The laser was focused and directed through a 20× antireflection-coated objective (LMU, NA: 0.5, UVB). The laser power was between 18 and 15 mW, leading to about 0.5 mW before the objective. The backscattered Raman light was redirected through a 400 μm entrance slit to a 2400 lines/mm grating and was detected by a nitrogen-cooled CCD camera (spectral resolution 2 cm^−1^). During the measurement, the sample was constantly rotated by a rotating stage in a spiral manner to avoid burning. Using a system of gears, the microscope table was pushed to the back while constantly rotating in order to create a spiral track of the laser beam on the sample. When the laser reached the edge of the sample, the gear system pushed the table back to the front, with a small offset to avoid measuring the same positions again. This way, a new spiral track was created parallel to the first one. Before each measurement, the sample was shifted on the stage to ensure the measurement of a different position each time. On every measurement day, 10 measurements were performed, consisting of 10 spectra × 15 s integration, which were later averaged. During data analysis, each time series was averaged into one spectrum. The measurement of the oligonucleotides and isolated DNA were performed as single measurements with 180 s excitation.

### 2.4. Data Preprocessing and Analysis

Data preprocessing was conducted using the Ramanmetrix software designed inhouse for Raman spectroscopic data analysis (RAMANMETRIX.eu, version 0.3.1, Jena, Germany) and using the Gnu R software (version 4.0.2, R Core Team, Vienna, Austria) [34] based on in-house developed scripts. A cosmic ray spike removal was conducted first [35]. For the VIS-spectra, the wavenumber axis was calibrated using the reference spectra of 4-acetylaminophenol [36], while the intensity calibration was performed using the standard material SRM2242 [37,38]. For the UV-spectra, the wavenumber calibration, which had a polynomial fit function, was based on polystyrene spectra that were collected on each measurement day. The baseline correction was performed using the sensitive nonlinear iterative peak (SNIP) clipping algorithm [39], with 40 iterations for both the VIS- and UV-spectra. To make the Raman spectra of individual bacterial cells comparable, the Raman intensity was standardized by vector normalization, where each spectrum was divided by its 2-norm (i.e., the square root of the sum of squared spectral intensities) [40]. The Raman spectra were then averaged for each isotope treatment.

## 3. Results and Discussion

### 3.1. Comparison of Isotope Uptake in Bacterial Cells

**Figure 1:**
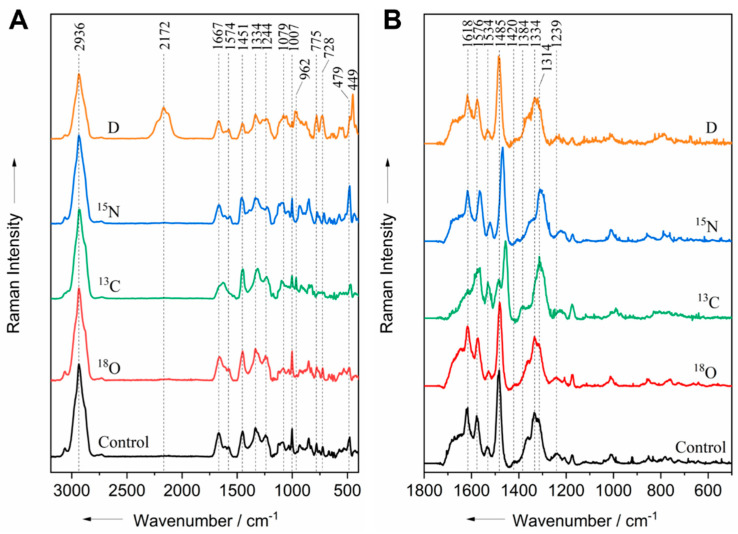
Raman spectra of isotopically labeled *E. coli* cells and unlabeled control acquired at the 532 nm (**A**) and 244 nm (**B**) excitation wavelengths.

*E. coli* cells were incubated for 24 h in the presence/absence of four different isotopes (^18^O, ^13^C, ^15^N, and D). The spectra acquired at the 532 and 244 nm excitation wavelengths are shown in Figure 1. The most prominent band observed in the 532 nm spectra at 2936 cm^−1^ is associated with the C–H stretching vibrations of all of the biomolecules (Figure 1A). As expected, this band is red-shifted to 2172 cm^−1^ in the spectrum of bacterial cells labeled with deuterium. The band at 2172 cm^−1^ is assigned to the C–D stretching vibration, indicating the substitution of hydrogen by deuterium in the cellular biomass [15] and therefore causing a decrease in the intensity of the C–H signal of the deuterium-labeled cells (Figure 1A). The Raman signal at 1667 cm^−1^ is attributed to the C=O stretching vibration of amide I (Figure 1A). This band is red-shifted to 1628 and 1656 cm^−1^ in ^13^C and ^18^O-labeled bacteria, respectively. However, it was expected that the red shift caused by ^18^O incorporation would be larger than that caused by ^13^C incorporation due to the larger mass difference between ^16^O/^18^O atoms compared to ^12^C/^13^C atoms. This observation shows that the ^16^O atoms in the C=O stretching vibration of the amide I group were only partially replaced by ^18^O; it might be that the metabolic pathway used by bacterial cells to metabolize H_2_^18^O is more restricted than that for ^13^C glucose. A detailed study of the red-shift that results from ^18^O uptake in the amide I band has been reported elsewhere [4]. Another feature of proteins is the band at 1244 cm^−1^, which arises from the C–N stretching vibration and the N–H bending mode of amide III (Figure 1A). The incorporation of ^13^C and ^15^N into bacterial cells resulted in a red-shift of the amide III signal to 1236 and 1232 cm^−1^, respectively. The red-shift caused by ^13^C substitution in amide III was smaller than the red-shift caused by ^15^N substitution because the latter involves both the C–N stretching vibration and the N–H bending mode. The ring breathing vibrations of phenylalanine and tryptophan result in a Raman signal at 1007 cm^−1^. This band was red-shifted to 962 cm^−1^ when the bacterial cells were incubated with ^13^C and D. These two band features have been observed previously when labeling with the same isotopes [15,19,28] and is likely related to a partial exchange of light and heavy atoms. In deuterium labeled cells, the band at 962 cm^−1^ appears when H/D exchange occurs at positions 1, 3, and 5 on the phenyl ring [15], whereas in ^13^C-labeled cells, the band at 962 cm^−1^ is attributed to a phenyl ring fully labeled with ^13^C [19].

The contribution of nucleic acids gives rise to the band at 1574 cm^−1^, which is due to the C=N stretching vibration of the purine bases (guanine and adenine), and the band at 728 cm^−1^ can be attributed to the ring breathing mode of adenine (Figure 1A). These bands are red-shifted to 1565 and 713 cm^−1^ in ^15^N-labeled bacterial cells, respectively. Deuterium incorporation into bacterial cells caused a red-shift of the adenine signal from 479 cm^−1^ to 449 cm^−1^ (Figure 1A); this red-shift is never reported when the bacteria are incubated with fully deuterated glucose. Our results from the 532 nm excitation are in agreement with the results presented by other authors [4,7,24,27] and can therefore be used as a reference to prove the isotopic labeling of biomolecules.

The UVRR spectra of *E. coli* cells labeled with various isotopes (^18^O, ^13^C, ^15^N, and D) are depicted in Figure 1B. The band around 1618 cm^−1^, which is attributed to the C=C stretching mode of aromatic amino acids (tyrosine, tryptophan, and phenylalanine), is red-shifted to 1576 cm^−1^ in the spectra of the ^13^C labeled cells. The peak at 1576 cm^−1^, which is associated with the combined C=C and C=N stretching vibrations of guanine and adenine [41], is red-shifted to 1563 cm^−1^ in the spectra of both ^13^C- and ^15^N-labeled cells. Another feature of adenine, located at 1420 cm^−1^ and assigned to the CH_2_-deformation vibration of adenine, is red-shifted to 1384 and 1405 cm^−1^ as a result of ^13^C and ^15^N incorporation, respectively (Figure 1B). The N9C8 and C8N7 stretching vibrations of the purine bases (guanine and adenine) give rise to the most prominent peak at 1485 cm^−1^ [42,43]. This peak is red-shifted to 1455 and 1470 cm^−1^ due to ^13^C and ^15^N assimilation in cells, respectively (Figure 1B). The cytosine signal at 1534 cm^−1^ exhibits a red-shift to 1521 cm^−1^ in the spectra of the ^15^N labeled cells (Figure 1B). Another characteristic band at 1334 cm^−1^ is assigned to the C−N stretching vibration of guanine, adenine, and tryptophan [41,44]. The incorporation of ^13^C and ^15^N into bacterial cells caused a red-shift of this band to 1314 and 1311 cm^−1^, respectively.

At 532 nm excitation, the assimilation of all of the isotopes was detected. When applying UVRR, no shift could be detected in the bacteria incubated with D. In addition, ^18^O labeling could also not be detected at 244 nm excitation because the assimilation occurred in the C=O stretching group of the amide I of proteins, which is not in resonance with the applied UV excitation wavelength [45]. Compared to all isotopes, deuterium uptake led to the largest red-shift in the Raman silent region of bacteria spectra in VIS. The red-shifts of ^13^C and ^15^N labeling in the VIS varied from 8 to 39 cm^−1^ and from 13 to 54 cm^−1^ in the deep-UV range. The observed red-shifts indicate that the isotopes were assimilated in both the proteins and nucleic acids of the bacterial cells. By using excitation wavelengths of 532 and 244 nm, different vibrational modes of the same molecules were observed, resulting the bacteria having a different spectral profile. A summary of the observed red shifts can be found in Table S1.

### 3.2. Raman Spectra of Oligonucleotides

**Figure 2:**
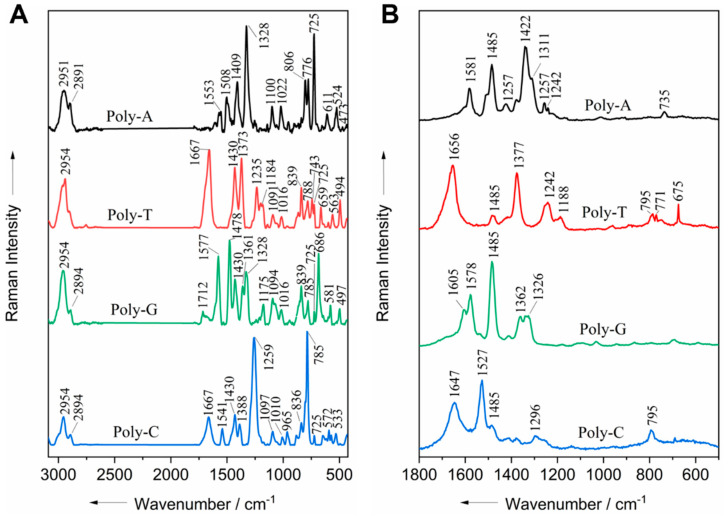
Raman spectra of oligonucleotides measured with visible excitation at 532 nm (**A**) and UVRR at 244 nm (**B**).

In order to confirm the band assignment of the characteristic bands of the nucleic acids as found in the literature and to adjust to the current calibration settings, the Raman spectra of the oligonucleotides consisting of a 10mer sequence of a one nucleotide base were acquired. The results from both wavelengths are shown in Figure 2. It can be observed that in the 532 nm spectra of all of the oligonucleotides, the vibrations of all of the DNA bases provide many Raman signals close to each other (Figure 2A). The band at 725 cm^−1^, which is associated with the ring breathing vibration of adenine appears, with high intensity in poly-A and with lower intensity in all of the other oligonucleotides. In contrast, only selectively enhanced signals appear in the 244 nm spectra (Figure 2B).

In the UVRR spectra, the band associated with the (N9C8 and C8N7) stretching vibrations along the long axis of the purine bases (guanine and adenine) [43] appears at 1485 cm^−1^, with high intensity for poly-A and poly-G and lower intensity for the poly-C and poly-T oligonucleotides (Figure 2B). Similar observations have been reported previously with pure nucleobases [46] and oligonucleotides of the same structure [33]. The peaks found in the spectrum of each oligonucleotide are summarized in Table S2. It was observed for all of the oligonucleotides that the spectra of both excitation wavelengths showed peaks at many similar positions (Table S3).

Small differences in the presence and position of bands are expected between single-stranded oligonucleotides and double-stranded DNA that have been extracted or contained in the cell due to the absence of H-bonds and base stacking in the single-stranded structure [33]. In addition, a decrease in the Raman intensity (hypochromatism) of certain functional modes in the UV region is expected when bases are stacked in double helix DNA due to an increase in structural complexity, which modifies the electron density of the DNA units and consequently their vibrational properties [33,47]. However, these differences in the spectrum are minor and should not affect the band assignment of bacteria and DNA samples, as performed in this work.

### 3.3. Isotope Accumulation in Nucleic Acids

**Figure 3:**
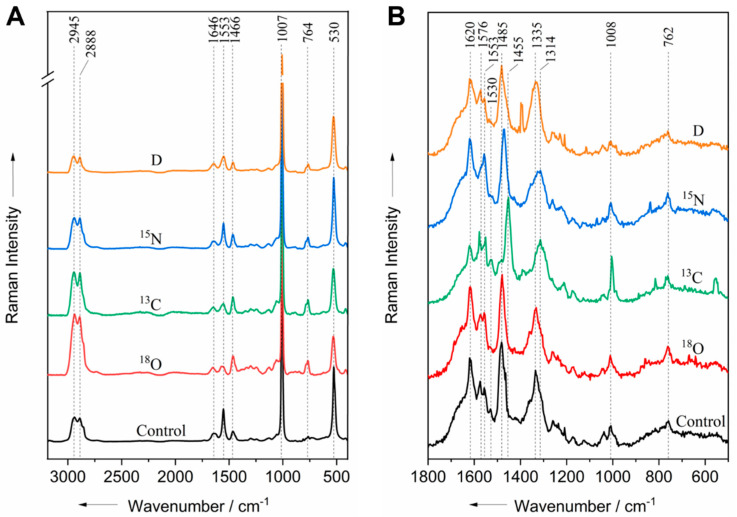
Raman spectra from DNA isolated from isotope-labeled bacterial cells and unlabeled control acquired at the 532 nm (**A**) and 244 nm (**B**) excitation wavelengths.

Raman microspectroscopy with an excitation wavelength in VIS provides information on the biochemical composition of the entire bacterial cell. In contrast, UVRR spectroscopy at 244 nm excitation can selectively enhance Raman signals from aromatic amino acids and nucleic acids. Due to the overlapping of protein and nucleic acid bands in the bacterial spectrum, it is difficult to understand isotope incorporation in detail. In order to better understand the assimilation profile of each isotope, Raman spectroscopy was performed on DNA extracted from the labeled bacteria. The Raman spectra acquired at both excitation wavelengths are depicted in Figure 3. At 532 nm excitation, Raman signals were observed at 1646, 1553, 1466, 764, and 530 cm^−1^ for all of the DNA-extracted samples.

An intense sharp peak is present in the spectra of all of the DNA samples at 1007 cm^−1^ (Figure 3A); this peak can be assigned to phosphate salts from the buffers used in the extraction procedure [48]. A similar peak profile was observed in the acquired buffer spectra (Figure S1). Therefore, the peaks observed in the 532 nm spectra were associated with the extraction buffers. At 532 nm excitation, the signal of the nucleobases at 728 cm^−1^, the adenine band at 479 cm^−1^, and the guanine and adenine band at 1574 cm^−1^ could not be detected in the DNA spectra of all of the treatments, including the control. Additionally, the C–D band of deuterium labeled cells was not detected (Figure 3A). Since no nucleic acids bands were observed at the 532 nm excitation wavelength, consequently, no red-shifts were detected in the spectra of the DNA extracts compared to the control. The reason for this is most likely that the Raman scattering cross-section of the nucleic acids at the 532 nm excitation wavelength is very small. Thus, the above observations indicate that the nucleic acid was successfully separated from other biomolecules. All of the isotopically labeled biomolecules detectable at 532 nm were removed from the sample during the extraction procedure. 

Unlike at 532 nm excitation, the Raman scattering cross-section of nucleic acids is significantly higher at the 244 nm excitation wavelength. Therefore, the investigation of the same DNA extracts with UVRR showed red-shifts in the spectra of DNA labeled with ^13^C and ^15^N. Similar to the bacterial cells, the guanine band at 1334 cm^−1^ shifted by 20 and 23 cm^−1^, and the purine band at 1485 cm^−1^ shifted by 30 and 15 cm^−1^ when labeled with ^13^C and ^15^N, respectively. Hence, the peaks at 1334 and 1485 cm^−1^ can serve as potential markers for the identification of ^13^C- and ^15^N-labeled cells using 244 nm excitation. A summary of the observed red-shifts can be seen in Table S4. Compared to the bacteria spectra (Figure 1B), only two red-shifts were observed in the spectra of the extracted DNA (Figure 3B); this is not surprising since the assimilation of the isotopes also occurred in aromatic amino acids, which were discarded during the DNA extraction procedure. The reduced number of red-shifts in DNA extracts indicates that isotope exchange occurred more in proteins than in nucleic acids; consequently, less functional groups of DNA labeled with isotopes were detected at an excitation wavelength of 244 nm.

An adenine peak that was not visible in the UVRR of bacterial cells was detected at 1553 cm^−1^ in the spectra of the DNA extracts. Differences were observed in the spectral profile of the extracted DNA compared to the spectral profile of bacterial cells. It has been previously shown that the Raman spectra of DNA differ significantly when measured inside the fixed cell nucleus and when extracted from the nucleus [49]. Additionally, the DNA spectrum of sperm cells differ significantly when measured inside the sperm head compared to when they are extracted [50]. This is due to structural changes occurring in the chromatin during the extraction procedure, taking into consideration that the presence of DNA-packing and supercoiling inside the cell does contribute to the vibrational profile of the molecule. DNA conformation is another factor contributing to these differences since the DNA of the living cell is a mixture of A, B, and Z DNA; each of these forms shows a distinct spectrum [51,52], whereas the extracted DNA only contains B DNA. These factors could explain the appearance of bands in the DNA spectrum that were not present or that were clearly visible in the spectrum of the bacterial cells. Additionally, small spectral differences are expected when comparing the double-stranded DNA present in the cells with single-stranded oligonucleotides.

Overall, the red-shifts observed in the UV spectra of extracted DNA are valuable proxies of the applicability of the presented approach to non-culturable samples. Indeed, this approach can overcome the limitations of the standard CO_2_ method and allow the identification of metabolically active cells in a rapid and non-destructive manner, while providing information on the biochemical composition of the bacteria, which gives a broad insight into the cellular activity in response to environmental changes.

## 4. Conclusions and Outlook

This study has demonstrated that UV resonance Raman spectroscopy is a powerful tool for the identification of isotopically labeled cells. Characteristic red-shifts were found in the spectra of isotopically labeled bacterial cells and in the spectra of DNA extracted from labeled cells. These results show that Raman-SIP in the deep-UV range can overcome the limitations of the VIS range, e.g., when studying ^15^N-labeled cells. Therefore, the red-shifts identified in this work are potential markers for monitoring the activity of bacterial cells in a given environment at an excitation wavelength of 244 nm. This is a very important result, demonstrating that the combination of isotope shift and the resonance effect of nucleic acids can serve as a useful tool for detecting metabolically active cells based on nucleic acid information. The combination of UVRR spectroscopy and stable isotope probing is therefore a promising approach for the direct screening of the genetic material of active bacterial cells when the cultivation of the bacteria is not possible. This could be performed by filtering out the bacteria from a sample followed by isotope labeling and detection of the red shifts in the spectra of the isolated genetic material of bacteria.

The present approach may offer opportunities for monitoring metabolic activity in complex environmental and clinical samples with low bacterial load. The results of this proof-of-concept study can be used as a basis for the development of new approaches to address the urgent need for identifying metabolically active cells in environmental microbiology and biomedical fields. One potential application is the detection of persistent cells, i.e., bacteria that are in a dormant or metabolically inactive state in tissues and exhibit tolerance to antibiotic treatments [53]. They occur in intercellular and extracellular environments, are present in all major pathogen groups, and play a central role in recurrent infections associated with or without biofilm formation, such as soft tissue infections, bone and joint infections, or pneumonia in cystic fibrosis patients [54,55]. This approach could also be applied in the detection of microbial load and VBNCs in water or food industry. Since the cultivation of bacteria in the sample is not mandatory, simple DNA extraction from a labeled sample can provide evidence for the presence of living bacteria. We believe that Raman-SIP in the deep-UV range can serve as a simple and rapid method for the detection of active or persistent bacterial cells.

## Figures and Tables

**Table 1:** Concentrations of DNA extracted from *E. coli* cells amended with different isotopes.

Isotope Treatment	$C_{\mathrm{DNA}}/\mathrm{ng}\;$µL^−1^ Batch 1	$C_{\mathrm{DNA}}/\mathrm{ng}\;$µL^−1^ Batch 2
H_2_O (control)	26.8	20.8
^18^O	25.2	32.6
^13^C	13.3	22.4
^15^N	23.0	21.6
D	8.6	14.4

## Data Availability

The data presented in this study are available upon request from the corresponding author.

## Supplementary Materials

The following are available online at https://www.mdpi.com/article/10.3390/life11101003/s1, Figure S1: Raman spectra of DNA extraction buffers, Table S1: Overview of band red-shifts in the Raman spectra of labeled bacterial cells, Table S2: Tentative band assignment of oligonucleotides, Table S3: Oligonucleotide peaks observed at both the 532 and 244 nm excitation wavelengths, Table S4: Overview of band red-shifts in the Raman spectra of labeled DNA.
