# Supplementary material for: Raman Stable Isotope Probing of Bacteria in Visible and Deep UV-Ranges
Source: Life (Basel). 2021 Sep 24;11(10):1003. doi: 10.3390/life11101003 (PMC8539138; doi:10.3390/life11101003)
Supplement: Supplementary file 1 [file life-11-01003-s001.zip › life-1382847-supplementary.pdf]

Supplementary Materials Raman Stable Isotope Probing of Bacteria in Visible and Deep UV-Ranges

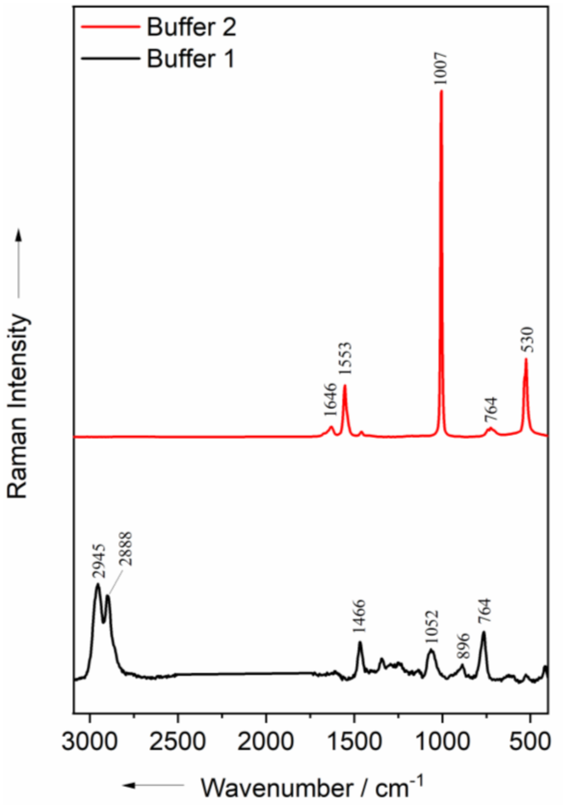

Figure S1. Mean Raman spectra of DNA extraction buffers.

Table S1. Red-shifts in the Raman bands of bacteria labeled with various isotopes. (/): new band red-shift observed in this work; no previous reference.

532 nm					
**Isotope**	**Raman band of unlabeled cells / cm^{-1}**	**Raman band of red-shift / cm^{-1}**	**Extent of Raman shift / cm^{-1}**	**Tentative assignement (Assignment from literature)**	**Reference for red-shift**
**D**	2936	2172	764	C–H stretching vibration of all biomolecules [1]	[2]
**^{18}O**	1667	1656	11	C=O stretching vibration of amide I (1663 cm^{-1}) [1,3]	[3-5]
**^{13}C**		1628	39		
**^{15}N**	1574	1565	9	C=N stretching vibration of guanine and adenine (1577 cm^{-1}) [6]	[3,5]
**^{13}C**	1244	1236	8	C–N stretching vibration of amide III (1241 cm^{-1}) [7]	[3,5]
**^{15}N**		1232	12		
**^{13}C**	1007	962	39	Phe ring breathing (1007 cm^{-1}) [3,8,9]	[3,5]
**D**		962	39		
**^{15}N**	728	713	12	Ring breathing of adenine (723 cm^{-1}) [3]	[3,5]
**D**	479	449	30	Adenine (473 cm^{-1})	/
**244 nm**					
**Isotope**	**Raman band of unlabeled cells / cm^{-1}**	**Raman band of red-shift / cm^{-1}**	**Extent of Raman shift / cm^{-1}**	**Tentative assignement (Assignment from literature)**	**Reference for red-shift**
**^{13}C**	1618	1564	54	C=C stretching vibration of Tyr, Trp, and Phe (1609 cm^{-1}) [10]	/
**^{13}C**	1576	1563	13	C=C and C=N stretching vibrations of guanine and adenine (1578 cm^{-1}) [10,11]	/
**^{15}N**		1563	13		
**^{15}N**	1534	1521	13	Cytosine (1533 cm^{-1}) [12]	/
**^{13}C**	1485	1455	30	N9C8 and C8N7 stretching vibrations along the long axis of purine bases (1485 cm^{-1})[13,14]	/
**^{15}N**		1470	15		
**^{13}C**	1420	1384	36	CH_2-deformation vibration of Adenine (1420 cm^{-1}) [15,16]	/
**^{15}N**		1405	15		
**^{13}C**	1334	1314	20	C–N stretching vibration of guanine, adenine, and tryptophan (1334 cm^{-1}) [11]	/
**^{15}N**		1311	23		

Table S2. Peaks from oligonucleotide spectrum from both excitation wavelengths. A: adenine, T: thymine, C: cytosine, G: guanine, (/): no peak appearance.

	532 nm			244 nm		
**Oligonucleotide**	**Band / cm^{-1}**	**Tentative assignement**	**Reference**	**Band / cm^{-1}**	**Tentative assignement**	**Reference**
**Poly-A**	473			735		
	524	Vibration of ribose phospate	[15]	1242		
	611			1257		
	725	Ring breathing vibration of adenine	[3,5,16]	1311		
	776			1422	CH_2-deformation vibration	[17]
	806	Ribose phosphodiester symmetric stretching	[15]	1485	N9C8 and C8N7 stretching vibrations along the long axis of purine bases	[13,14]
	1022			1581	NH_2 deformation virbration	[14]
	1100	PO_2-streching vibration	[17]	/	/	/
	1328	C5N7, N7C8 stretching vibration	[14]	/	/	/
	1409	C4N9, C8H deformation vibration	[13]	/	/	/
	1508			/	/	/
	1553			/	/	/
	2891	CH_2 symmetric streching vibration	[18]	/	/	/
	2951	CH_2 asymmetric streching vibration	[18]	/	/	/

	532 nm			244 nm		
**Oligonucleotide**	**Band / cm^{-1}**	**Tentative assignement**	**Reference**	**Band / cm^{-1}**	**Tentative assignement**	**Reference**
**Poly-T**	494			675		
	563	Vibration of ribose phospate	[15]	771		
	659	Ribose phosphodiester symmetric stretching	[15]	795	PO_2 symmetrick streching vibration	[17]
	725	Ring breathing vibration of adenine	[3,5]	1188	C-C and C-N stretching vibration	[14]
	788	PO_2 symmetric streching vibration	[17]	1242	C6H deformation vibration, C4N4	[13]
	839	O—P—O streching vibration	[17]	1377	C6H bending vibration, ring stretching viration	[14]
	1016	Vibration of ribose	[15]	1485	N9C8 and C8N7 stretching vibrations along the long axis of purine bases	[13,14]
	1091	PO_2-streching vibration	[17]	1656	C4=O-C4C5 streching vibration	[13,14]
	1184	C-C and C-N stretching vibration	[14]	/	/	/
	1235			/	/	/
	1373	C6H bending vibration	[14]	/	/	/
	1430	CH_2-deformation vibration	[17]	/	/	/
	1667	C=O stretchichng vibration	[16]	/	/	/
	2954	CH_2 asymmetric streching vibration	[18]	/	/	/

	532 nm			244 nm		
**Oligonucleotide**	**Band / cm^{-1}**	**Tentative assignement**	**Reference**	**Band / cm^{-1}**	**Tentative assignement**	**Reference**
**Poly-C**	533			795	PO_2-streching vibration	[17]
	572			1296		
	725	Ring breathing vibration of adenine	[3,5]	1485	N9C8 and C8N7 stretching vibrations along the long axis of purine bases	[13,14]
	785	PO_2 symmetric streching vibration, Ring breathing vibration	[13,16]	1527		
	836	O—P—O streching vibration	[17]	1647	C2=O stretching vibration	[13]
	965		[17]	/	/	/
	1010	Vibration of ribose phospate	[15]	/	/	/
	1097	PO_2-streching vibration	[13]	/	/	/
	1259	C6H deformation vibration, C4N4	[17]	/	/	/
	1388	C4N, N2C2 stretching vibration	[13]	/	/	/
	1430	CH_2-deformation viration	[14]	/	/	/
	1541	N3=C4 vibration	[17]	/	/	/
	1667	C=O stretching	[13]	/	/	/
	2894	CH_2 symmetric streching vibration	[18]	/	/	/
	2954	CH_2 asymmetric streching vibration	[18]	/	/	/

	532 nm			244 nm		
**Oligonucleotide**	**Band / cm⁻¹**	**Tentative assignement**	**Reference**	**Band / cm⁻¹**	**Tentative assignement**	**Reference**
**Poly-G**	497	Vibration of ribose phospate	[15]	1328	C–N stretching vibration	[11,13]
	581	Vibration of ribose phospate	[15]	1362	N7C8, N1C6, N5N7 stretching vibration	[14]
	686	Ring breathing vibration	[16]	1485	N9C8 and C8N7 stretching vibrations along the long axis of purine bases	[13,14]
	725	Ring breathing vibration of adenine	[3,5]	1578	NH_2 deformation vibration	[14]
	785	PO_2-streching vibration	[13,16]	/	/	/
	839	O–P–O stretching vibration	[16]	/	/	/
	1016	Vibration of ribose		/	/	/
	1094	PO_2-streching vibration	[17]	/	/	/
	1175	C-C and C-N streching vibration	[15]	/	/	/
	1328	C5N7, N7C8 stretching vibration	[14]	/	/	/
	1361	N7C8, N1C6, N5N7 stretching vibration	[14]	/	/	/
	1430	CH_2-deformation vibration	[17]	/	/	/
	1478	N9C8 and C8N7 stretching vibrations along the long axis of purine bases	[13,14]	/	/	/

	532 nm			244 nm		
**Poly-G (continued)**	1577	NH_2 deformation vibration	[14]	/	/	/
	1712	C6=O stretch	[13]		/	
	2894	CH_2 symmetric streching vibration	[18]	/		/
	2954	CH_2 asymmetric streching vibration	[18]	/	/	/

Table S3. Peaks from oligonucleotide spectrum that appear at both excitation wavelengths. A: adenine, T: thymine, C: cytosine, G: guanine, (/): no peak appearance.

A		T		C		G	
**532 nm**	**244 nm**	**532 nm**	**244 nm**	**532 nm**	**244 nm**	**532 nm**	**244 nm**
725 cm^{-1}	735 cm^{-1}	1184 cm^{-1}	1188 cm^{-1}	785 cm^{-1}	795 cm^{-1}	1328 cm^{-1}	1326 cm^{-1}
/	/	1235 cm^{-1}	1242 cm^{-1}	1667 cm^{-1}	1647 cm^{-1}	1361 cm^{-1}	1362 cm^{-1}
/	/	1667 cm^{-1}	1656 cm^{-1}	/	/	1478 cm^{-1}	1485 cm^{-1}
/	/	/	/	/	/	1577 cm^{-1}	1578 cm^{-1}

Table S4 Red-shifts in the UV Raman bands of of DNA isolated from bacteria incubated with isotopes.

Isotope	Raman band of unlabeled cells / cm^{-1}	Raman band of red-shift / cm^{-1}	Extent of Raman shift / cm^{-1}	Assignement
**^{13}C**	1334	**1314**	20	C–N stretching vibration of guanine, adenine, and tryptophan
**^{15}N**		**1311**	23	
**^{13}C**	1485	**1455**	30	N9C8 and C8N7 stretching vibrations along the long axis of purine bases
**^{15}N**		**1470**	15	

References

1. Harz, M.; Rösch, P.; Popp, J. Vibrational spectroscopy—A powerful tool for the rapid identification of microbial cells at the single-cell level. *Cytometry A* **2009**, *75A*, 104-113, doi:doi:10.1002/cyto.a.20682.

2. Tao, Y.; Wang, Y.; Huang, S.; Zhu, P.; Huang, W.E.; Ling, J.; Xu, J. Metabolic-Activity-Based Assessment of Antimicrobial Effects by D2O-Labeled Single-Cell Raman Microspectroscopy. *Anal. Chem.* **2017**, *89*, 4108-4115, doi:10.1021/acs.analchem.6b05051.

3. Huang, W.E.; Li, M.; Jarvis, R.M.; Goodacre, R.; Banwart, S.A. Chapter 5 - Shining Light on the Microbial World: The Application of Raman Microspectroscopy. In *Advances in Applied Microbiology, Vol 70*, Laskin, A.I., Sariaslani, S., Gadd, G.M., Eds.; Advances in Applied Microbiology; Academic Press: 2010; Volume 70, pp. 153-186.

4. Azemtsop Matanfack, G.; Pistiki, A.; Rösch, P.; Popp, J. Raman 18O-labeling of bacteria in visible and deep UV-ranges. *Journal of Biophotonics 14*, e202100013, doi:10.1002/jbio.202100013.

5. Wang, Y.; Huang, W.E.; Cui, L.; Wagner, M. Single cell stable isotope probing in microbiology using Raman microspectroscopy. *Curr. Opin. Biotechnol.* **2016**, *41*, 34-42, doi:10.1016/j.copbio.2016.04.018.

6. Žukovskaja, O.; Kloß, S.; Blango, M.G.; Ryabchykov, O.; Kniemeyer, O.; Brakhage, A.A.; Bocklitz, T.W.; Cialla-May, D.; Weber, K.; Popp, J. UV-Raman Spectroscopic Identification of Fungal Spores Important for Respiratory Diseases. *Anal. Chem.* **2018**, *90*, 8912-8918, doi:10.1021/acs.analchem.8b01038.

7. Kloß, S.; Kampe, B.; Sachse, S.; Rösch, P.; Straube, E.; Pfister, W.; Kiehntopf, M.; Popp, J. Culture Independent Raman Spectroscopic Identification of Urinary Tract Infection Pathogens: A Proof of Principle Study. *Anal. Chem.* **2013**, *85*, 9610-9616, doi:10.1021/ac401806f.

8. Wang, Y.; Song, Y.; Tao, Y.; Muhamadali, H.; Goodacre, R.; Zhou, N.Y.; Preston, G.M.; Xu, J.; Huang, W.E. Reverse and Multiple Stable Isotope Probing to Study Bacterial Metabolism and Interactions at the Single Cell Level. *Anal. Chem.* **2016**, *88*, 9443-9450, doi:10.1021/acs.analchem.6b01602.

9. Kirchhoff, J.; Glaser, U.; Bohnert, J.A.; Pletz, M.W.; Popp, J.; Neugebauer, U. Simple Ciprofloxacin Resistance Test and Determination of Minimal Inhibitory Concentration within 2 h Using Raman Spectroscopy. *Anal. Chem.* **2018**, *90*, 1811-1818, doi:10.1021/acs.analchem.7b03800.

10. Walter, A.; Reinicke, M.; Bocklitz, T.; Schumacher, W.; Rösch, P.; Kothe, E.; Popp, J. Raman spectroscopic detection of physiology changes in plasmid-bearing Escherichia coli with and without antibiotic treatment. *Anal. Bioanal. Chem.* **2011**, *400*, 2763-2773, doi:10.1007/s00216-011-4819-4.

11. Silge, A.; Heinke, R.; Bocklitz, T.; Wiegand, C.; Hipler, U.-C.; Rösch, P.; Popp, J. The application of UV resonance Raman spectroscopy for the differentiation of clinically relevant Candida species. *Anal. Bioanal. Chem.* **2018**, *410*, 5839-5847, doi:10.1007/s00216-018-1196-2.

12. Walter, A.; Schumacher, W.; Bocklitz, T.; Reinicke, M.; Rösch, P.; Kothe, E.; Popp, J. From Bulk to Single-Cell Classification of the Filamentous Growing Streptomyces Bacteria by Means of Raman Spectroscopy. *Appl. Spectrosc.* **2011**, *65*, 1116-1125, doi:10.1366/11-06329.

13. Neugebauer, U.; Schmid, U.; Baumann, K.; Simon, H.; Schmitt, M.; Popp, J. DNA tertiary structure and changes in DNA supercoiling upon interaction with ethidium bromide and gyrase monitored by UV resonance Raman spectroscopy. *J. Raman Spectrosc.* **2007**, *38*, 1246-1258, doi:10.1002/jrs.1760.

14. Harz, M.; Krause, M.; Bartels, T.; Cramer, K.; Rösch, P.; Popp, J. Minimal Invasive Gender Determination of Birds by Means of UV-Resonance Raman Spectroscopy. *Analytical Chemistry* **2008**, *80*, 1080-1086, doi:10.1021/ac702043q.

15. Tu, A. *Raman Spectroscopy in biology*; Wiley-International: USA, 1982.

16. Benevides, J.M.; Overman, S.A.; Thomas Jr, G.J. Raman, polarized Raman and ultraviolet resonance Raman spectroscopy of nucleic acids and their complexes. *J. Raman Spectrosc.* **2005**, *36*, 279-299, doi:10.1002/jrs.1324.

17. Neugebauer, U.; Clement, J.H.; Bocklitz, T.; Krafft, C.; Popp, J. Identification and differentiation of single cells from peripheral blood by Raman spectroscopic imaging. *Journal of Biophotonics* **2010**, *3*, 579-587, doi:10.1002/jbio.201000020.

18. Azemtsop Matanfack, G.; Taubert, M.; Guo, S.; Bocklitz, T.; Küsel, K.; Rösch, P.; Popp, J. Monitoring Deuterium Uptake in Single Bacterial Cells via Two-Dimensional Raman Correlation Spectroscopy. *Anal. Chem.* **2021**, *93*, 7714-7723, doi:10.1021/acs.analchem.1c01076.
